# Epidemiology, outcomes, and access to care for pediatric patients who underwent surgery in Northern Tanzania: A cross-sectional study

**DOI:** 10.1371/journal.pgph.0004448

**Published:** 2025-06-27

**Authors:** Pamela Espinoza, Theresia Mwakyembe, Happiness D. Kajoka, Prisca Joseph Stephano, Esther Majaliwa, Blandina T. Mmbaga, Catherine Staton, Joao Ricardo Nickenig Vissoci, Henry E. Rice, Emily R. Smith

**Affiliations:** 1 Duke Global Health Institute, Duke University, Durham, North Carolina, United States of America; 2 Global Emergency Medicine Innovation and Implementation Research Center, Duke University, Durham, North Carolina, United States of America; 3 Department of Surgery, Kilimanjaro Christian Medical Centre, Moshi, Tanzania; 4 Kilimanjaro Christian Medical University College, Moshi, Tanzania; 5 Pediatric Hematology and Oncology Services, Kilimanjaro Christian Medical Centre, Moshi, Tanzania; 6 Kilimanjaro Clinical Research Institute, Kilimanjaro Christian Medical Centre, Moshi, Tanzania; 7 Department of Emergency Medicine, Duke University Medical Center, Durham, North Carolina, United States of America; 8 Division of Pediatric Surgery, Department of Surgery, Duke University Medical Center, Durham, North Carolina, United States of America; Yale University School of Medicine, UNITED STATES OF AMERICA

## Abstract

Lack of access to surgical care is common in low- and middle-income countries, where children and adolescents account for up to half of the population. However, the burden of surgical conditions and resources for children in Tanzania remains poorly defined. Our cross-sectional study aims to assess the epidemiology and outcomes of pediatric surgical procedures performed over one year at the Kilimanjaro Christian Medical Centre (KCMC), a tertiary center in Moshi, Tanzania. A secondary aim was to assess the geospatial distribution of families seeking surgical care and their accessibility to surgical services at KCMC. We evaluated the surgical records of all children <16 years old who underwent surgical care between January 1^st^, 2022 and December 31^st^, 2022 at KCMC. We used descriptive statistics to compare demographic and clinical characteristics across age groups. Geospatial mapping tools were used to visualize families’ district of origin and access to care. We collected data on 2031 children, 59.8% (n = 1215) of whom were male, and more than half were between 1 and 5 years old (58.6%, [n = 1191]). Families traveled from 28 out of the 31 Tanzanian regions. Overall, about half of the conditions were diseases of the respiratory system (52.9%, [n = 1074]), followed by congenital and developmental disorders (14.6%, [n = 297]) and injuries (8.4%, [n = 170]). We identified 33 (1.6%) deaths prior to discharge. Children under one year old had lower rates of insurance coverage, higher referral rates, traveled longer distances, and had worse clinical outcomes compared to other age groups (p < 0.001). Our study reveals differences in the demographic and clinical characteristics of children who access specialized surgical care at KCMC. We report a high number of elective ear, nose, and throat (ENT) procedures and low number of non-ENT procedures, suggesting barriers to care for acute, high morbidity conditions.

## Introduction

Surgical care is a foundational element of a well-functioning health system and plays a crucial role in global pediatric health [1–4]. For children, surgical care is key to treat a wide spectrum of conditions that range from congenital conditions to injuries and cancer [4–7]. Surgical procedures are critical to reduce morbidity and mortality rates among children worldwide [8]. It is estimated that 1.7 billion children and adolescents around the world lack access to safe and affordable surgical care when needed [1]. Most of these children live in low- and middle-income countries (LMICs), where 1.1 billion children do not have access to surgical care [1,2,9].

In Tanzania, 19% of deaths and 17% of Disability-Adjusted Life Years (DALYs) have been attributed to surgically treatable conditions [10]. About 43% of the population is less than 15 years old and thus, making tailored plans to address the surgical needs of children is necessary [11]. The referral pathway for surgical services in Tanzania begins at primary health facilities (primary level) and district hospitals (secondary level) [10,12]. However, only 18% of primary health centers perform surgical procedures, and district hospitals often report shortages of qualified personnel and adequate infrastructure to perform common surgical procedures for children, such as pediatric hernia repairs [10,13]. For this reason, pediatric surgical patients are often referred from lower-level facilities to tertiary level facilities such as regional, zonal, and national hospitals to receive care [12,14]. The Kilimanjaro Christian Medical Centre (KCMC) is one of the two hospitals in the country dedicated to pediatric surgery [15]. As a zonal referral hospital with a catchment population of over 11 million people, KCMC treats a wide range of surgical conditions for children from across the nation [15,16].

To date, only a limited number of studies in Tanzania have focused on surgical care, generally for selected surgical conditions such as trauma or congenital anomalies in children [17–19]. Understanding the surgical needs for children is critical not only for understanding the burden and distribution of pediatric surgical disease, but also for policy guidance for allocating pediatric health resources. This study aims to assess the epidemiology and outcomes of all pediatric surgical procedures performed over one year at KCMC. A secondary aim was to assess the geospatial distribution of families seeking surgical care and their accessibility to surgical services at KCMC. These findings can inform current national efforts to strengthen the provision of surgical care in the country and advocate for the creation of resilient pediatric surgical systems in Tanzania.

## Materials and methods

### Study design, period, and setting

This cross-sectional study evaluated medical and surgical records of all children who underwent surgery between January 1^st^, 2022 and December 31^st^, 2022 at KCMC in Moshi, Tanzania. Tanzania is divided into 31 regions and 170 districts and has a population of over 61 million people [20,21]. Tanzania’s gross domestic product per capita is $1192, classifying it as a lower-middle income country according to the 2022 World Bank income classification [22]. Approximately 35% of the population lives in rural areas, and about 32% is covered by any type of health insurance [23,24].

The Lancet Commission on Global Surgery (LCoGS) has proposed six core indicators to assess a country’s progress in achieving safe and affordable surgical care, including the goal of 5000 surgical procedures per 100,000 population performed annually and a surgical, anesthetic, and obstetric (SAO) physician density of 20 per 100,000 population [2,25]. In Tanzania, only 484 surgical procedures are performed per 100,000 population annually and the SAO density is 0.46 per 100,000 population [25]. In addition, a previous assessment of pediatric surgical capacity at district level hospitals in Tanzania showed that surgical services were mostly limited to minor emergency and elective procedures in wound care, male circumcision, and fracture management [13]. Complex congenital conditions (e.g., Hirschsprung’s disease or oesophageal atresia) were typically not handled at this level and were referred to higher-level centers like regional or zonal hospitals. Systemic challenges have been reported in the referral system including infrastructure and workforce limitations [10,13,26]. Most surgical procedures on children at the district level were reported to be delivered by general medical officers rather than general or pediatric surgeons [13]. Similarly, nurse anesthetists not anesthesiologists provided anesthesia in 92% of the district hospitals surveyed [13]. Thus, the most common reasons for surgical referrals in the country are due to staff or infrastructure limitations [12,14]. Prior to 2018, there was no official training for specialist pediatric surgeons in the country and to address this gap, a pediatric surgery program at Muhimbili National Hospital (MNH) in Dar es Salaam was implemented [27]. While efforts to increase the pediatric surgical workforce have been put in place, specialists are often only available in urban areas and higher-level hospitals like KCMC.

KCMC is located in Northern Tanzania and is part of a health complex that includes the Kilimanjaro Clinical Research Institute and Kilimanjaro Christian Medical University College [16]. The hospital has five main operating theaters: general surgery, emergency surgery, gynecology, orthopedic surgery, and septic surgery [28]. In addition, KCMC has specialized theaters for the ear, nose, and throat (ENT), ophthalmology, urology, and dermatology departments. The average number of children receiving surgery in the main operating theaters is two per day. However, this number can increase in case of emergencies. Specialized theaters such as the ENT and ophthalmology theaters have a capacity of up to five and four pediatric surgeries per day, respectively. The pediatric surgery ward has a total of 18 beds while the neonatal intensive care unit (NICU) has six beds. General surgeons and other specialist surgeons for adults perform most surgical procedures on children. As a result, many children are referred to receive surgical care at this tertiary level hospital. Medical and surgical records are stored in an electronic medical record system officially put in place in 2019.

### Source population and study participants

KCMC serves as a key referral hospital for over 4 million children under 15 years old in Northern Tanzania [29,30]. In this study, we used a convenience sample of all children who underwent surgery at KCMC during 2022 and included all patients who were 15 years old or younger at the time of the operation [31]. No exclusion criteria were applied.

### Variables and data collection

Prior to data collection, two experienced research assistants received training on the study protocol and on how to access and manage de-identified data. We collected de-identified demographic and clinical information from medical records, nurses’/doctors’ notes, anesthesiology reports, and surgical operative reports. All data were available in the electronic medical record and collected between June 1^st^, 2023 and September 15^th^, 2023. The research assistants accessed the electronic file of each patient and collected de-identified data. The data was then securely stored in a Research Electronic Data Capture (REDCap) database. During the data collection period, we conducted periodic quality checks and discussed any discrepancies. At the end of the data collection, we conducted a final review to ensure the completeness and accuracy of the data.

#### Demographic variables.

Demographic variables included age, sex, district and region of origin, primary caregiver, and insurance status. We report our results in age cohorts as follows: < 1 year old, 1–5, 6–10, and 11 – 15. The caregiver accompanying the child during the hospital visit was recorded as the primary caregiver. Insurance status included insured, defined as coverage through the National Health Insurance Fund (NHIF), other insurance mechanisms, international aid programs and charities, or uninsured.

#### Clinical variables.

Clinical variables included diagnosis based on the International Classification of Diseases, 10^th^ Revision (ICD-10), surgical procedure, urgency of procedure (elective/emergency), referral information, duration of symptoms, pre-operative status using the American Society of Anesthesiologists (ASA) score, type of anesthesia, time between hospital arrival and surgery, length of hospitalization, and outcome of surgery [32,33].

The ICD-10 codes were assigned by the hospital staff at the time of the surgical procedure and recorded in the patient’s surgical operative report. The clinical research team reviewed cases with multiple ICD-10 codes, with the diagnosis more causally related to the surgical need assigned as the primary diagnosis. Diagnoses were grouped based on the broader ICD-10 chapters.

We recorded the name of the surgical procedure and urgency of the operation from the patient’s surgical operative report and the provider’s notes. Elective surgeries were defined as those that were scheduled or conditions that did not require immediate treatment. Emergency procedures were those that required immediate surgical treatment and/or entered the hospital through the emergency department.

The referral information on the last facility visit and the duration of symptoms in days were obtained from the provider’s notes (reported in months). Since neither variable was systematically recorded in the medical records, they were only obtained whenever they were available.

The classification for type of anesthesia was obtained from the anesthesiology reports, where it was defined as local, regional, or general. The ASA scores, also available in the anesthesiology reports, ranged between 1 (normally healthy patient) and 5 (a life-threatening condition that needs immediate surgery) and were collapsed into the following levels for analysis purposes: 1, 2, and equal to or greater than 3 [33].

#### Clinical outcomes.

The post-surgical outcomes were defined as discharge with no complications, complications, and death prior to discharge. Complications included surgical site infections or when a second surgery was needed due to complications of the first surgery as identified in the clinical record. Perioperative death was defined if the child died after the surgery or during the hospital admission. No data on surgical outcomes after the discharge were available. The time in hours between hospital arrival and surgery was estimated using the timestamp found in the admission record and the cut time for the surgery (reported in days). The length of hospitalization was estimated by counting days from the admission date to the discharge date.

#### Geospatial variables.

To estimate the distance between the patient’s district of origin and KCMC, the total roadway travel distance in kilometers and time in minutes between the district’s centroid and KCMC was calculated using the ArcGIS Online tool. The tool used fixed speeds obtained from historical average car speed data for Tanzania [34]. The street data were obtained from the 2023Q3 update and provided by ESRI [34,35]. The regional and district level data represent the sub-national distribution of Tanzania in 2019, accounting for the addition of the Songwe region in 2016 [20]. The basemap was provided by ESRI and the Tanzania districts layer provided by Tanzania National Bureau of Statistics and United Nations OCHA [20,36].

### Statistical analysis

Descriptive statistics were used to assess the distribution of demographic and clinical characteristics across all patients and also stratified by age groups. Categorical variables were described using proportions, and quantitative variables were summarized using median and interquartile range (IQR). Children’s age groups were compared across demographic and clinical characteristics using analysis of Kruskal-Wallis, Fisher Exact Test or Chi-squared test of independence when applicable. Associations were considered statistically significant at p < 0.05. The number of missing values for each variable is presented in the results. All analyses were conducted using R (version 4.2.1; R Core Team 2021). This study complies with the Strengthening the Reporting of Observational Studies in Epidemiology (STROBE) statement (S1 Appendix).

### Ethical considerations

Ethical approval was received from the KCMC Institutional Review Board (Protocol 2576), National Institute for Medical Research (NIMR) in Tanzania (Protocol NIMR/HQ/R.8a/Vol.IX/4066), and the Duke University Institutional Review Board (Protocol 00110763). No personal health information was collected in this study, and all data are presented at an aggregate level. Therefore, informed consent was not required by the aforementioned institutional review boards.

## Results

### Demographics

Over 2022, 2031 children between 0 and 15 years of age received surgery at KCMC (**Table 1**). Of these 2031 patients, 59.8% (n = 1215) were male and 40.2% (n = 816) were female. More than half of the children were between 1 and 5 years old (58.6%, [n = 1191]). The next most common age group was 6–10 years old (21.0%, [n = 426]). Overall, over two-thirds of the patients were insured (70.1%, [n = 1424]). However, for the youngest and oldest age groups, 22.8% (n = 39) and 59.3% (n = 144) of the children were insured, a lower rate compared to the middle age groups, which showed insurance coverage greater than 70% (p < 0.001).

**Table 1:** Demographic and clinical characteristics of pediatric surgical patients by age group at KCMC, Tanzania (n = 2031).

	Total	Age groups (years)				
		< 1	1 – 5	6 – 10	11 – 15	P value
**Total,** n (%)	2031 (100)	171 (8.4)	1191 (58.6)	426 (21.0)	243 (12.0)	NA
**Demographic characteristics**						
**Sex,** n (%)						
Male	1215 (59.8)	91 (53.2)	720 (60.5)	247 (58.0)	157 (64.6)	0.1014^a^
Female	816 (40.2)	80 (46.8)	471 (39.5)	179 (42.0)	86 (35.4)	
**Kilimanjaro Region,** n (%)						
No	646 (31.8)	80 (46.8)	314 (26.4)	146 (34.3)	106 (43.6)	<0.001^a^
Yes	1385 (68.2)	91 (53.2)	877 (73.6)	280 (65.7)	137 (56.4)	
**Distance (km)**						
Median (IQR)	28 (5 – 100)	92 (20 – 213)	20 (5 - 92)	28 (5 - 100)	88 (5 - 249)	<0.001^b^
Missing, n	29	7	10	7	5	
**Caregiver,** n (%)						
Mother/Father	1648 (88.4)	143 (88.8)	993 (90.4)	330 (85.9)	182 (82.7)	0.0040^a^
Other	216 (11.6)	18 (11.2)	106 (9.6)	54 (14.1)	38 (17.3)	
Missing	167	10	92	42	23	
**Insurance status,** n (%)						
Insured	1424 (70.1)	39 (22.8)	937 (78.7)	304 (71.4)	144 (59.3)	<0.001^a^
Uninsured	607 (29.9)	132 (77.2)	254 (21.3)	122 (28.6)	99 (40.7)	
**Clinical characteristics**						
**ICD-10 classification,** n (%)						
Congenital and developmental disorders	297 (14.6)	106 (62.0)	136 (11.4)	37 (8.7)	18 (7.4)	<0.001^a^
Diseases of the digestive system	114 (5.6)	31 (18.1)	53 (4.5)	15 (3.5)	15 (6.2)	
Diseases of the eye and adnexa	139 (6.8)	5 (2.9)	44 (3.7)	48 (11.3)	42 (17.3)	
Diseases of the respiratory system	1074 (52.9)	9 (5.3)	791 (66.4)	207 (48.6)	67 (27.6)	
Injury, poisoning & external causes	170 (8.4)	6 (3.5)	68 (5.7)	59 (13.8)	37 (15.2)	
Neoplasms	79 (3.9)	6 (3.5)	32 (2.7)	19 (4.5)	22 (9.1)	
Other	158 (7.8)	8 (4.7)	67 (5.6)	41 (9.6)	42 (17.3)	
**Referral,** n (%)						
No	829 (70.3)	34 (29.3)	540 (79.8)	176 (70.7)	79 (57.2)	<0.001^a^
Yes	351 (29.7)	82 (70.7)	137 (20.2)	73 (29.3)	59 (42.8)	
Missing	851	55	514	177	105	
**Duration of symptoms (months)**						
Median (IQR)	6 (1 - 24)	1 (0 - 3)	12 (2 - 24)	6 (0 - 24)	6 (0 - 24)	<0.001^b^
Missing, n	576	19	361	125	71	
**Type of procedure,** n (%)						
Elective	1910 (94.0)	144 (84.2)	1147 (96.3)	391 (91.8)	228 (93.8)	<0.001^a^
Emergency	121 (6.0)	27 (15.8)	44 (3.7)	35 (8.2)	15 (6.2)	
**ASA score,** n (%)						
1	641 (36.7)	17 (11.5)	388 (37.5)	133 (36.6)	103(51.2)	<0.001^a^
2	1016 (58.2)	111 (75.0)	605 (58.5)	215 (59.2)	85 (42.3)	
≥ 3	90 (5.2)	20 (13.5)	42 (4.1)	15 (4.1)	13 (6.5)	
Missing	284	23	156	63	42	
**Anesthesia type,** n (%)						
Local/ regional	70 (3.4)	3 (1.8)	19 (1.6)	11 (2.6)	37 (15.2)	<0.001^a^
General	1961 (96.6)	168 (98.2)	1172 (98.4)	415 (97.4)	206 (84.8)	
**Time between arrival and surgery,** n (%)						
< 1 day	765 (37.7)	72 (42.1)	449 (37.7)	156 (36.6)	88 (36.2)	<0.001^a^
1 – 3 days	1079 (53.1)	43 (25.1)	667 (56.0)	243 (57.0)	126 (51.9)	
4 – 6 days	96 (4.7)	26 (15.2)	39 (3.3)	19 (4.5)	12 (4.9)	
> 7 days	91 (4.5)	30 (17.5)	36 (3.0)	8 (1.9)	17 (6.9)	
**Length of stay (days)**						
Median (IQR)	3 (3 - 6)	8 (5 - 16)	3 (3 - 4)	3 (3 - 5)	5 (3 - 8)	<0.001^b^
**Outcome of surgery,** n (%)						
Discharge with no complications	1960 (96.5)	141 (82.5)	1161 (97.5)	419 (98.4)	239 (98.4)	<0.001^c^
Complications	38 (1.9)	9 (5.3)	21 (1.8)	5 (1.2)	3 (1.2)	
Deaths prior to discharge	33 (1.6)	21 (12.3)	9 (0.8)	2 (0.5)	1 (0.4)	

IQR = interquartile range; ICD-10 = International Classification of Diseases, 10th Revision; ASA = American Society of Anesthesiologists; NA = not applicable; KCMC = Kilimanjaro Christian Medical Centre.

^a^ Chi-square test of independence;

^b^ Kruskal-Wallis rank sum test;

^c^ Fisher exact test.

Overall, about half of the surgical conditions were diseases of the respiratory system (52.9%, [n = 1074]), followed by congenital and developmental disorders (14.6%, [n = 297]) and injury, poisoning & other consequences of external causes (8.4%, [n = 170]). The most common procedures within each disease category were tonsillectomies and adenoidectomies within diseases of the respiratory system (96.6%), ventriculoperitoneal shunts within congenital and developmental disorders (17.8%), open reduction internal fixation (ORIF) within injury, poisoning & external causes (25.3%), hernia repairs within diseases of the digestive system (53.5%), and biopsies and tumor excisions within neoplasms (22.8%) (**Table 2**). Overall, there were 38 cases with complications and 33 in-hospital mortality cases, accounting for 1.9% and 1.6% of the surgical procedures, respectively in 2022.

**Table 2:** Top ten most common pediatric surgical procedures overall and top three by disease group at KCMC (n = 2031).

Surgical procedure	n (%)
**Overall (n = 2031)**	
Tonsillectomy/adenoidectomy	1037 (51.1)
Laparotomy	69 (3.4)
Ventriculoperitoneal shunt	64 (3.2)
Hernia repair	63 (3.1)
Open reduction internal fixation (ORIF)	44 (2.2)
Biopsy	38 (1.9)
Cataract removal with and without intraocular insertion	38 (1.9)
Foreign body removal	35 (1.7)
Surgical debridement	34 (1.7)
Lens washout with intraocular lens insertion	34 (1.7)
**Diseases of the respiratory system (n = 1074)**	
Tonsillectomy/adenoidectomy	1037 (96.6)
Turbinectomy	21 (2.0)
Sinusectomy	5 (0.5)
**Congenital and developmental disorders**^**a**^ **(n = 297)**	
Ventriculoperitoneal shunt	53 (17.8)
Hypospadias repair	29 (9.8)
**Injury, poisoning & external causes (n = 170)**	
Open reduction internal fixation (ORIF)	43 (25.3)
Foreign body removal	34 (20.0)
Surgical debridement	22 (12.9)
**Diseases of the eye and adnexa (n = 139)**	
Corneal grafting, vitrectomy, retinal, orbital, and ocular correction surgery	28 (20.1)
Cataract removal with and without intraocular insertion	27 (19.4)
Lens washout with intraocular lens insertion	14 (10.1)
**Diseases of the digestive system**^a^ **(n = 114)**	
Hernia repair	61 (53.5)
Laparotomy	38 (33.3)
**Neoplasms (n = 79)**	
Biopsy	18 (22.8)
Tumor excision	18 (22.8)
Eye enucleation	11 (13.9)
**Diseases of the genitourinary system (n = 44)**	
Catheter Insertion	8 (18.2)
Urethroplasty	8 (18.2)
Cystoscopy	5 (11.4)
**Diseases of the musculoskeletal & connective tissue (n = 39)**	
Surgical debridement	11 (28.2)
Contracture release	5 (12.8)
Abscess incision & drainage	4 (10.3)
**Diseases of the ear and mastoid process (n = 31)**	
Myringotomy and grommets insertion	22 (71.0)
Tympanoplasty	5 (16.1)
Examination under anesthesia	2 (6.5)
**Diseases of the nervous system (n = 11)**	
Ventriculoperitoneal shunt	7 (63.6)
External ventricular drainage	3 (27.3)
Burr hole	1 (9.1)
**Diseases of the skin and subcutaneous tissue (n = 10)**	
Abscess incision & drainage	4 (40.0)
Fistula excision	2 (20.0)
Cyst excision	2 (20.0)
**Other**^b^ **(n = 23)**	
Cauterization of bleeding vessel	4 (17.4)

KCMC = Kilimanjaro Christian Medical Centre. Notes: Because this table only reflects the top three most common surgical procedures per disease group, the sum of percentages per group will not equal one hundred percent.

^a^ Multiple conditions tied for third most common procedure.

^b^ Multiple procedures tied for second and third most common procedures.

### Surgical characteristics by age

The most common surgical conditions for children under 1 year were congenital and developmental disorders (62%, n = 106) (**Table 1**). Among children 1 – 5 years old, surgical procedures related to the respiratory system were the most common type of procedure (66.4%, [n = 791]). Although diseases of the respiratory system were the most frequent conditions for children 6 – 10 years old (48.6%, [n = 207]), the percentage of injury, poisoning & other consequences of external causes and diseases of the eye and adnexa were higher compared to the younger age groups. The percentage of neoplasm cases was highest among children 11 – 15 years old (9.1%, [n = 22]).

The clinical characteristics for children under 1 year differed the most from other age groups. The duration of symptoms was the shortest and the emergency procedure rate was the highest among this age group. Among children under 1 year old, 13.5% (n = 20) had an ASA score greater than 3, with lower rates of high ASA scores in other age groups. The median hospital stay for children under 1 year was 8 days (IQR: 5 – 16) while the median stay for the rest of the age groups ranged from 3 – 5 days. When looking at the patients who died prior to discharge, 21 out of 33 cases were children under 1 year.

### Geospatial distribution of families seeking surgical care

**Fig 1:**
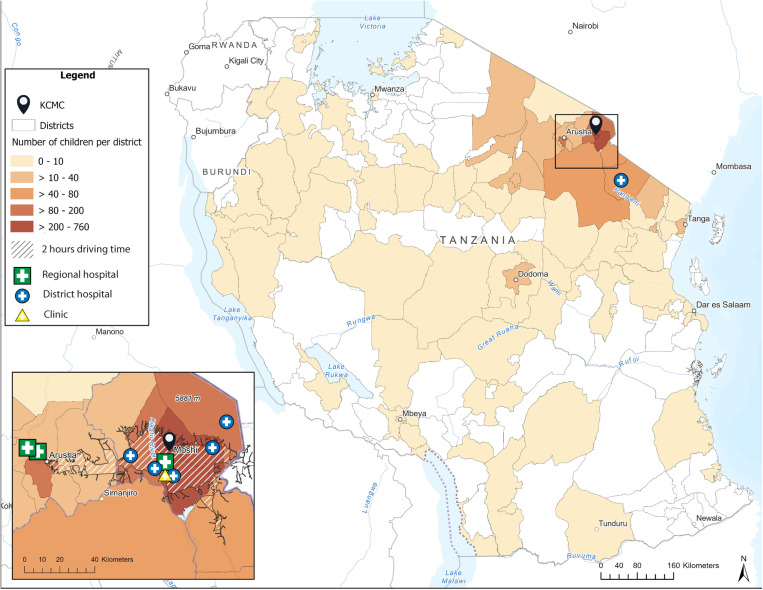
Geospatial distribution of pediatric surgical patients at the Kilimanjaro Christian Medical Center based on district of origin (n = 2031). Notes: About 56.8% of children who received surgery at KCMC lived within two hours (hatched area on map). Regional and district hospitals on the map represent the ten most common referral facilities in this study. Basemap provided by ESRI (https://www.arcgis.com/home/item.html?id=2ef1306b93c9459ca7c7b4f872c070b9).

Children and their families traveled from 28 out of the 31 Tanzanian regions to receive surgical care at KCMC (**Fig 1**). The median travel distance was 28 km (IQR: 5 – 100). However, the youngest and oldest age groups traveled greater distances compared to the middle age groups. The median travel distance for children under 1 year old and 11–15 years old was 92 km (IQR, 20 – 213) and 88 km (IQR, 5 – 249), respectively. Overall, 56.8% (n = 1154) of the children came from districts that were within a 2-hour driving time of KCMC.

**Fig 2:**
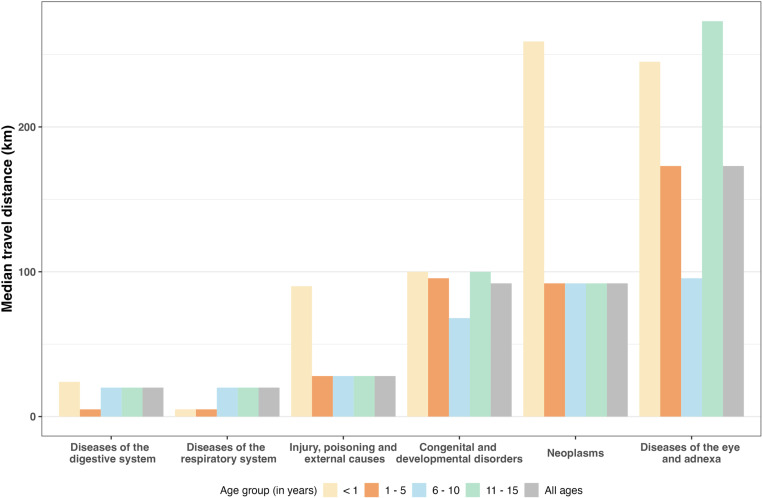
Median distance traveled by families to reach pediatric surgery by age and condition.

Geographic differences also existed depending on the type of surgery children received. The top three disease groups with the longest distance traveled were: diseases of the eye and adnexa (173 km, IQR: 88–546), neoplasms (92 km, IQR: 28–338), and congenital and developmental disorders (92 km, IQR: 20–273), (**Fig 2**). When stratifying by age, we observed a stark difference in children under 1 year old. Specifically, children under 1 year with neoplasms and injuries traveled distances three times the median distance for these conditions in all age groups.

### Referral patterns

**Fig 3:**
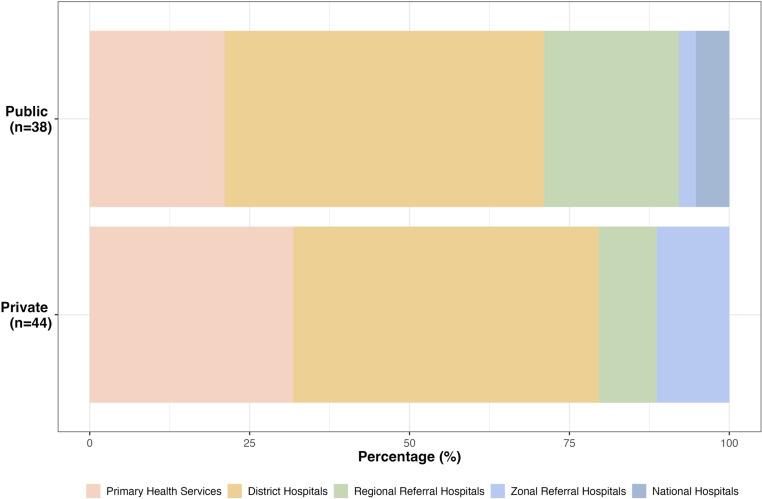
Type and level of referral facilities (n = 82).

Out of the 82 referral facilities, 38 (46.3%) were public and 44 (53.7%) were private (**Fig 3**). Most referrals came from primary health services and district hospitals, composing 71.1% of the public facilities and 79.5% of the private facilities. Only a small number of the referrals came from higher facilities such as zonal referral and national hospitals. In the under 1 year old age group, 70.7% (n = 82) of the children were referred from another health facility. However, the percentage of children referred from other facilities in older age groups were much lower and ranged from 20.2% to 42.8%.

## Discussion

A critical step towards achieving equitable access to pediatric surgical care is understanding where the current disparities exist [1,2]. Our study reveals a high burden of surgical needs in children and that many children in Tanzania travel long distances for surgical care, contributing to disparities in surgical care across the country. Children under one year old are particularly vulnerable, showing the lowest insurance coverage and longest travel distances. The findings of this one-year review are essential to inform ongoing national efforts aimed at reducing disparities and improving access to surgical care.

The LCoGS highlights the need to have access to essential surgical care within 2 hours [2]. In our study, about half of the children had no access to surgery within 2 hours and it is likely that this number is underestimated, since our estimate is based on the fastest route and access to a car. In Tanzania, car ownership is only 4.8% in urban areas and as low as 1% in rural areas [37]. A study at MNH in Dar es Salaam found that the median travel distance for children needing surgery was 4 hours [14]. Together with the results from our study, this finding highlights the geographic concentration of pediatric surgical expertise in urban areas and raises concerns about equitable and timely access to care in the country. Geography and lack of surgical services in rural areas have been described as main barriers to care in other studies across sub-Saharan Africa [38–41]. Particularly, a study in Uganda found that among children with untreated surgical conditions, 79% lived in rural areas while 21% lived in urban areas [42].

Ear, nose, and throat (ENT) procedures were by far the most common procedures performed in our study, yet data from the Global Burden of Disease shows that chronic respiratory diseases only accounted for about 0.95% of the total DALYs for children under 5 years old in Tanzania in 2021 [43]. As a comparator, congenital anomalies and injuries together made up 14.6% of the DALYs for the same age group in 2021 [43]. At other tertiary centers in neighboring countries, surgeries for congenital anomalies and injuries were the most common procedures for children [44,5]. Yet, surgeries for congenital anomalies and injuries were far less common than ENT procedures in our study. Such a high number of ENT procedures compared to other types of care suggests that there are significant access barriers for non-ENT care. However, an assessment of the unmet surgical needs and care at lower-level facilities would provide a comprehensive understanding of the surgical burden and service provision in the region.

Overall, 70.1% of the children who received surgery at KCMC were insured. However, for children under one year old, insurance coverage was 22.8%, lower than adults receiving surgery at KCMC (45.5%), children with emergency surgery at MNH (33.3%), and the Tanzanian average (32%) [14,24,28]. As well, children under one needed an urgent procedure more often and had worse clinical outcomes than the older age groups. In LMICs, fear of the costs of surgery, particularly among the uninsured, has been reported as a main reason to delay seeking care, increasing the chances of needing urgent care and worse outcomes [40–42]. In addition to having the lowest insurance rate, this age group had the highest referral rates and traveled the longest distances suggesting that families likely spent longer navigating the health system and incurring OOP costs. Financial protection for this vulnerable, uninsured population is critical [45–47]. By defining an essential pediatric surgical package and integrating it into existing maternal and child health plans, health leaders can leverage existing expertise to implement these programs in the country [48–50].

A significant portion of the children were referred from lower-level facilities, highlighting the importance of strong surgical systems and establishing standards of pediatric surgical resources at each level of care. For example, the “Optimal Resources for Improving Care” guide LMICs on the surgical resources required at each hospital level [51]. While surgical resources are critical, it is equally important to emphasize systemness—ensuring a careful calibration of service allocation, tailored to the health system’s strength, with contextual adaptation for each site [2,51]. Lastly, support at the community level is equally important. Such efforts can be carried out by community health workers (CHWs), who can address cultural and societal barriers to surgical care [52]. Training for CHWs should focus on the early identification of surgical conditions and referral of patients to the appropriate level of care. Finally, due to their proximity to the communities, CHWs can establish long-term relationships with families and assist with rehabilitation needs after surgery, supporting them throughout their entire surgical journey.

### Limitations

The primary limitations of this study are those inherent to single-institution retrospective studies. Although KCMC is a central institution providing pediatric surgical care, our study only captures those children who were able to receive surgical care at this hospital. For this reason, we cannot draw conclusions on the actual burden of disease or outcomes observed at other facilities/regions and might introduce some bias in the subgroup analysis and conclusions drawn from it. Similarly, since we used the surgical operative reports to conduct our review, we did not have data of children admitted with surgical conditions but that did not receive surgery. This introduces selection bias as we likely excluded patients that arrived at the hospital but could not afford to pay for surgery, arrived with worse conditions, or died prior to surgery. In addition, we recorded outcomes only to the point of discharge which might be a reason for the low number of post-surgery complications and deaths reported in this study. Due to limitations during data collection, further details on post-operative complications, cause of death, and classification of cases into minor/major were not obtained, which may limit our understanding of factors associated with poor outcomes. We also acknowledge that there are a number of parameters other than distance/time to reach the hospital that determine access to care not included in this study. Lastly, the only available data related to socioeconomic status was the patients’ insurance status. Thus, exact direct and indirect costs for receiving the surgery and household finances were not available, and conclusions regarding the impact of the surgery on families’ finances could not be drawn.

### Conclusion and recommendations

Our study provides an overview of all surgeries performed on children at one of the two tertiary hospitals dedicated to pediatric surgery in Tanzania. Children under one year old had the worst clinical outcomes and were more likely than other age groups to be uninsured, require urgent procedures, or have been referred to KCMC. Future efforts should focus on ensuring timely access to care and developing interventions to provide financial risk protection for these families. As well, we report a high number of elective ENT procedures and low number of non-ENT procedures. Extending this work to lower-level facilities and community level will be critical to have a complete picture of the current surgical need and strengthen the current provision of pediatric surgical services in the country. Lastly, interventions to address the geospatial disparities and increase access to surgical care in rural areas should be a high priority in the country.

## Supporting information

S1 Appendix STROBE checklist. (DOCX)
